# Complete chloroplast genome sequence of an orchid hybrid *Cymbidium sinense* (♀) × *C. goeringii* (♂)

**DOI:** 10.1080/23802359.2020.1839367

**Published:** 2020-11-20

**Authors:** Hong-Il Choi, Jae Il Lyu, Hyun-Oh Lee, Jin-Baek Kim, Sang Hoon Kim

**Affiliations:** a Advanced Radiation Technology Institute, Korea Atomic Energy Research Institute, Jeongeup, Republic of Korea; b Phyzen Genomics Institute, Seongnam, Republic of Korea

**Keywords:** Orchidaceae, orchid hybrid, *Cymbidium sinense* × *C. goeringii*, chloroplast genome

## Abstract

The complete chloroplast genome sequence of the *Cymbidium* hybrid, *C. sinense* (♀) × *C. goeringii* (♂) was assembled in this study. The circular genome was 150,149 bp in length with an overall GC content of 37.1% and consisted of a pair of 25,691 bp inverted repeats, and two single-copy regions that were 84,987 bp and 13,780 bp, respectively. Gene annotation analysis identified 109 genes including 75 protein-coding genes, 30 transfer RNA, and 4 ribosomal RNA genes. Phylogenetic analysis showed its closest relationship to *Cymbidium sinense*, reflecting a maternal inheritance of chloroplasts.

The genus *Cymbidium*, belonging to Orchidaceae, includes over 50 species of terrestrial, lithophytic, and epiphytic orchids from mountainous regions of tropical and subtropical Asia and northern Australia (Du Puy et al. 2007; Yang et al. 2013). Changing climate and habitat deterioration have led several orchid species to endangerment and even extinction, such as *C. goeringii*, whose wild populations have declined drastically over the past decades and is currently considered an endangered species (Wu et al. 2010; Park et al. 2018). Because of its esthetic value, *C. goeringii* is widely cultivated as an ornamental plant and new *C. goeringii* cultivars with different floral traits have been generated using classical- or mutational-breeding tools. Another way to retain the gene pool of an endangered species is to create hybrids, which can then be selected based on their traits.

The *Cymbidium* hybrid plant used in this study, *C. sinense* (♀) × *C. goeringii* (♂), was kindly provided by the orchid breeding company, Saemangeum Bio Center, Gimje, Republic of Korea (35°49′33.0″N, 126°51′38.0″E). Its botanical identity was confirmed and the plant germplasm, including rhizomes and DNA, have been deposited in Radiation Breeding Research Center, Advanced Radiation Technology Institute, Korea Atomic Energy Research Institute, Jeongeup, Republic of Korea with an accession number RB012 (Kim et al. 2020). Total genomic DNA was isolated from plant leaves and 300 bp paired-end (PE) sequenced using the Illumina MiSeq platform. Raw PE reads of about 1.9 Gb were trimmed (Phred scores of 20 or less) and high quality PE reads of about 1.2 Gb were subjected to *de novo* assembly using the CLC assembly cell package (v. 4.21, CLC Inc., Denmark), as described by Kim et al. (2015). Contigs containing chloroplast (CP) genome sequences were selected, ordered, and compared with the known CP genome sequence of *C. sinense* (NCBI accession number NC_021430) (Yang et al. 2013) to generate a single draft sequence. The draft sequence was manually corrected and gap-filled by mapping a series of PE reads to generate a complete CP genome sequence. The sequence was annotated using GeSeq (https://chlorobox.mpimp-golm.mpg.de/geseq-app.html).

The CP genome sequence of the *Cymbidium* hybrid was 150,149 bp in length and contained a large single-copy (SC) region of 84,987 bp and small SC region of 13,780 bp. Large and small SC regions were separated by a pair of inverted repeats (IR) that spanned 25,691 bp. The CP genome contained 109 genes, which included 75 protein coding genes, 30 transfer RNA, and four ribosomal RNA genes. Only 16 genes contained one (*atp*F, *pet*B, *pet*D, *rpl*2, *rpl*16, *rpo*C1, *rps*16, *trnA*-UGC, *trnG*-UCC, *trnl*-GAU, *trnK*-UUU, *trnL*-UAA, *trnV-*UAC) or two (*clp*P, *rps*12, *ycf*3) introns. Although the overall GC content was 37.1%, IR region showed a higher GC content (43.7%), whereas large and small SC regions contained 34.44% and 29.7% GC content, respectively.

Phylogenetic tree was constructed for 22 Orchidaceae CP genomes using maximum-likelihood (ML) method and bootstrap with 1000 times iteration using the MEGA7 program (Kumar et al. 2016) (Figure 1). The CP genome of the *Cymbidium* hybrid showed the closest relationship to that of previously reported *C. sinense* (NCBI reference sequence ID NC_021430, 155,548 bp in length) (Yang et al. 2013), reflecting a maternal inheritance of CPs. Sequence alignment using MAFFT (https://mafft.cbrc.jp/alignment/server/) and identification of polymorphisms between the two CP genome sequences of the *Cymbidium* hybrid and *C. sinense* revealed 37 single nucleotide polymorphisms and 50 insertions and deletions (InDels), scattered throughout the genome. Of these, two large InDels in the small SC region, 3.1 kb and 2.3 kb in length, respectively, contributed to the length difference between the two CP genomes. These may be derived from intraspecific genetic divergence by geographical isolation.

**Figure 1.**
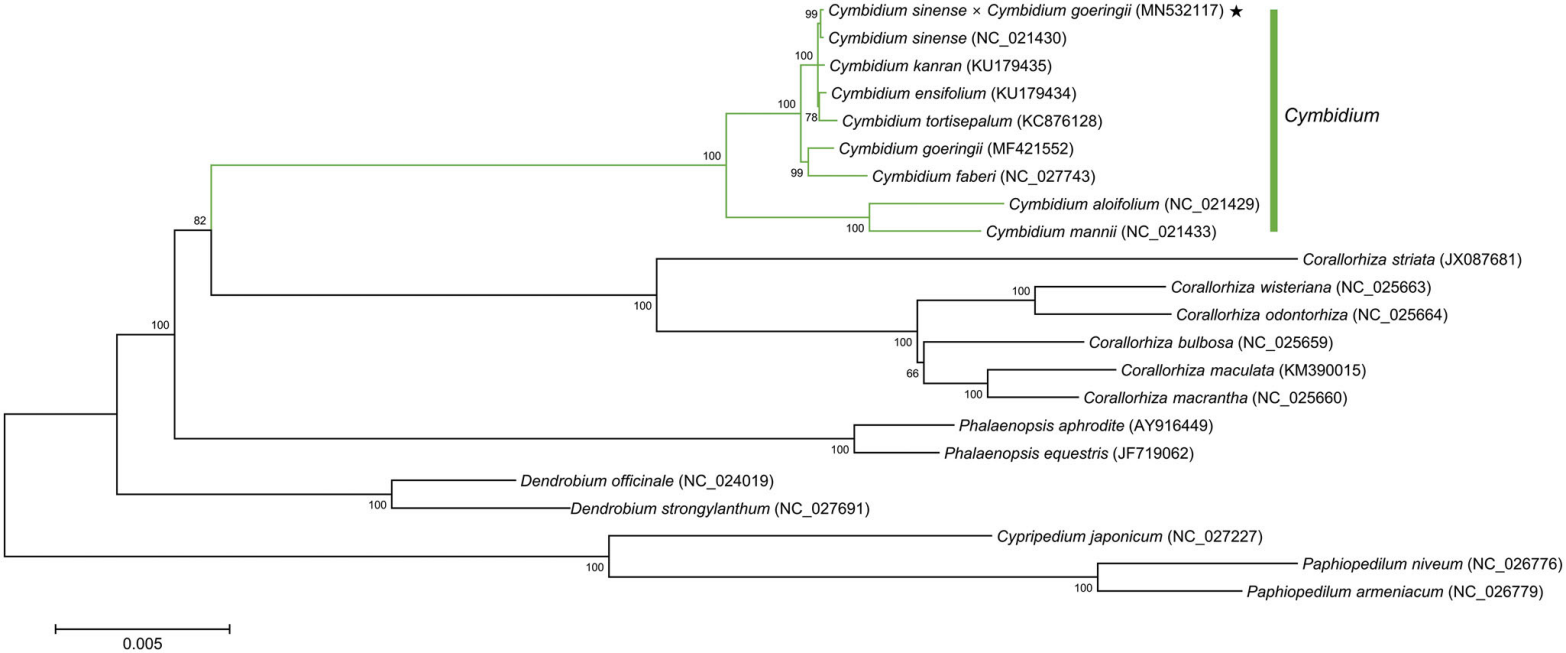
Maximun likelihood phylogenetic tree inferred from chloroplast genome sequences of 22 orchid species. Numbers on nodes indicate bootstrap values. Black star indicates the *Cymbidium* hybrid sequenced in this study.

## Data Availability

The data that support the findings of this study are openly available in NCBI (https://www.ncbi.nlm.nih.gov) GenBank with the accession number MN532117.
